# Use of Computer-Based Scenarios for Clinical Teaching: Impact on Nursing Students’ Decision-Making Skills

**DOI:** 10.3390/healthcare9091228

**Published:** 2021-09-17

**Authors:** Nermine M. Elcokany, Amal Ismael Abdelhafez, Vivian Magdi Samuel Sharaby, Safia Belal

**Affiliations:** Department of Nursing, College of Applied Medical Sciences, King Faisal University, Al-Ahsa 31982, Saudi Arabia; aabdelhameed@kfu.edu.sa (A.I.A.); vsharaby@kfu.edu.sa (V.M.S.S.); salsoudany@kfu.edu.sa (S.B.)

**Keywords:** computer-based simulation, clinical teaching, decision-making skills

## Abstract

Computer-based learning has numerous advantages. It gives students the chance to accommodate and solve problems independently, it can increase motivation during the learning process, and it offers students direct feedback. Students will also receive an authentic learning experience, increasing their level of knowledge retention. It can assist nursing educators in improving learning outcomes. Aim: This study aimed to investigate and evaluate the impact of computer-based scenarios on undergraduate nursing students’ decision-making skills. Sample: There was a total sample of 112 nursing students who were enrolled in a critical care nursing course at the College of Applied Medical Sciences in Saudi Arabia. These students were divided into two groups. Methods: The two groups were taught the same topic for one week. Two case scenarios were given to each group during the clinical rotation. The study group used the computer-based case scenario, and the control group used the paper-based case scenario. The two groups were compared regarding their decision-making skills. The student’s feedback about the computer-based case scenarios was also investigated. Results: The study group scored significantly higher in their decision-making skills when compared to the control group. In addition, the study group reported that they highly agreed that their general learning and specific nursing abilities improved after using computer-based case scenarios.

## 1. Introduction

Nurse educators are being required to integrate excellent teaching practices in the classroom and to guarantee that nursing students are motivated and engaged as a result of technological advancements. Technology can expose students to clinical scenarios they would not encounter in clinical practice so they can bridge the gap between theory and practice. We can add reality to a scenario and make traditional case studies more realistic using technology. Technology in the classroom can take different forms based on its definition [1]. Through computer-based learning, students will interact directly and independently with the computer. “Computer-based learning is a technique, using technology to simplify real experiences with guided experiences and considered one of the simulations that can be used in clinical teaching” [2]. Computer-based learning contains the dependable description of case scenarios using simulated realistic patient conditions. Those case scenarios require accurate, precise, and on-time decisions from the learners. To simulate those dependable clinical experiences, instructional different multimedia visualizations, such as animated characters, are used [3,4].

Computer-based learning has numerous advantages as it affords chances for students to accommodate and solve problems independently, it can raise the students’ motivation during the learning process and give direct feedback, students will authenticate learning experience, and the level of knowledge retention will be increased [2,5].

The education of nursing students needs the combination of intellectual and psychomotor skills to achieve the required competencies for practice. Intellectual skills include clinical reasoning and decision-making abilities, which demand knowledge of the circumstances. The development of situation awareness requires a comprehension of the current condition of a situation, which affects the decision-making process. Nurses employ a variety of patient data to better facilitate situation awareness, including patient diagnoses, an assessment of the data collected, and prediction of potential patient outcomes to help in care planning [6]. Critical care units are fast-paced, rapidly changing, and unstructured, and the patient status can deteriorate suddenly. Critical care nursing students need to be proficient in setting priorities for patient care and making effective decisions on time to ensure that care is managed efficiently according to patient needs [7]. Critical thinking skill development is multidimensional and covers numerous dimensions of metacognitive self-awareness. These include the ability to adapt to diverse and complex situations, the ability to apply theory into practice, and to show self-motivation and enterprising behaviors, strategic thinking, and an accurate assessment of one’s strengths and weaknesses [8].

Adequate time is needed to let the students engage in clinical practice. Students in clinical placement do not have consistency in caring for the patients, and this usually differs significantly from day to day based on the patient’s status and condition. For that, some students are exposed to clinical learning opportunities than others, which explains the variation among nurses in clinical practice skills [3,9]. Currently, many nursing institutions are using computer-based learning in clinical nursing education to encourage their students’ clinical reasoning, decision-making abilities, and clinical insight. Hence, it is one of the ideal tools for clinical skill improvement [10,11,12,13,14,15].

According to a study conducted by Alconero-Camarero, A.R. et al. (2021) [16], clinical simulation is more useful in many forms, such as computer-based scenarios. It provides students with an atmosphere conducive to the development of non-technical abilities. It is seen as a safe and controlled method since it allows them to make errors and correct them in real-time. Additionally, it is an effective clinical learning tool that does not threaten patient safety [16]. Computer-generated software is widely utilized in medical and nursing schools, with a variety of software packages that are effective at developing psychomotor skills and knowledge as well as clinical reasoning [4].

### Theoretical Framework

Within the constructivist paradigm, computer-based learning enhances student engagement and empowers students to have optimal control over their learning. As a method of achieving this, technology seeks to maximize the authentication of the learning context available, with the goal of minimizing the gap between classroom-acquired knowledge and the real-world situations that a nurse will encounter throughout their professional career. To optimize the learning experience and boost persistence with technology-based learning aids, studies have demonstrated that increased motivation, a favorable attitude toward such media, and positive self-efficacy are critical. Previous research has demonstrated that increasing a learner’s positive self-efficacy improves a learner’s ability to solve real-world challenges, which is correlated with increased accomplishment and retention [17,18,19,20].

**Figure 1:**
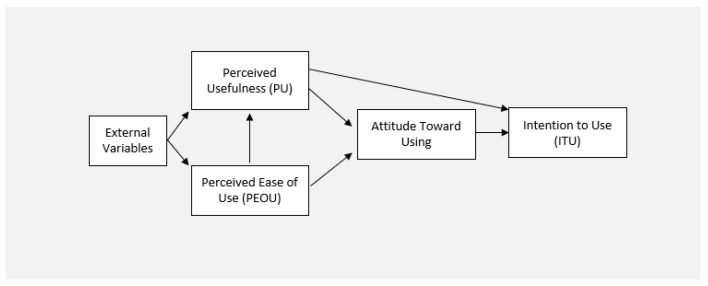
Technology acceptance model. Reprinted from (Huang 2018) [18].

User perceptions of technology-based materials can be determined by examining ease of use, application, utility, system, and information quality, as well as a user’s level of enjoyment. According to the technology acceptance model (TAM) (Figure 1), perceived utility and simplicity of use affect both the learners’ attitude toward future technology use and, more significantly, their performance [21].

Computer-based simulation activities provide a variety of opportunities for formative and summative assessment and help students improve their performance throughout the nursing program. A study conducted by Arrogante et al. (2021) found that students’ level of confidence and satisfaction with practical abilities increased when the simulation was used as a formative assessment method [22]. Additionally, computer-based learning has been linked to significant improvements in interpersonal communication skills, crisis management, and team performance in a variety of clinical situations. Additionally, it facilitates the effective development of transformational leadership skills and assists students in developing their self-efficacy and confidence in their clinical talents. The King Faisal University nursing program seeks to improve its students’ decision-making skills throughout the program, implementing computer-based programs as teaching strategies [23,24,25,26,27,28]. Therefore, this study was conducted to evaluate the impact of computer-based scenarios on undergraduate nursing students’ decision-making skills. 

## 2. Research Questions

(a) What are the scores for the students’ decision-making skills using computer-based and paper-based scenarios?
(b) What is the students’ feedback regarding computer-based scenarios?

## 3. Materials and Methods

### 3.1. Research Design

A quasi-experimental non-equivalent control group research design was used to achieve the aim of the current study.

### 3.2. Setting

This study was conducted at the College of Applied Medical Sciences (CAMS), Nursing Department, King Faisal University (KFU), Al Ahsa, Kingdom of Saudi Arabia. 

### 3.3. Subjects 

A total sample of 112 female nursing students enrolled in a level four critical care nursing course. All of them were Saudi nationality students, were in the age range of 20–23 years old, agreed to participate in the current study, and had no previous experience in computer-based case studies. This batch of students was comprised of two groups. The study group (78 students) was subjected to computer-based scenarios, whereas the control group (34 students) was subjected to paper-based scenarios. 

### 3.4. Tools

Two tools were used for data collection after extensively reviewing the relevant literature [3,28,29,30]. 

#### 3.4.1. Tool I: “Decision Making Skills Worksheet”

**Figure 2:**
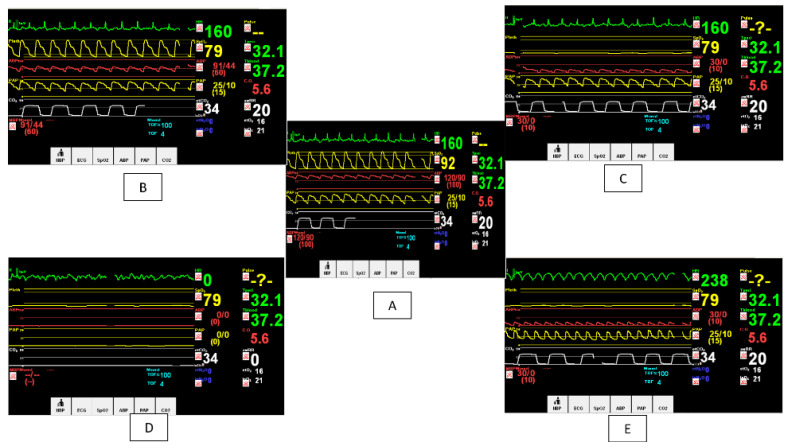
Screenshots of the software used with different hemodynamic changes in computer-based case study. (**A**) Atrial fibrillation with normal blood pressure and hypoxia; (**B**) Atrial fibrillation with hypoxia and hypotension; (**C**) Atrial fibrillation with hypoxia and hypotension; (**D**) Pulseless electrical activity with hemodynamic instability; (**E**) Ventricular tachycardia with hemodynamic instability).

This worksheet was developed for the students to record their answers after completing the case studies. The worksheet was composed of a blank table representing five different changes in temperature, heart rate, respiratory rate, oxygen saturation (SpO_2)_, mean arterial pressure (MAP), end-tidal carbon dioxide (etCO_2_), and type of dysrhythmia (Figure 2). It also included three open-ended questions about each type of dysrhythmia and the priority of managing them. 

The three parameters for evaluating this worksheet were:

1. A knowledge score with a grade given out of 25 (5 marks for each phase).
2. The priority of the action: 5 marks for the overall priority of managing the symptoms according to their risk of fatality.
3. The speed of response was difficult to determine, especially for the traditional group, so it was decided to evaluate the overall time taken by each student to submit the case study, given a total time of 30 min as the average criterion. One grade was deducted for each one-minute time delay, given a grade out of 10.

The total score of the worksheet ranged from 0 to 30 points. The students’ grades were categorized as follows: (0) poor, for those who scored less than 50% (15/30); (1) satisfactory, for those who scored from 50% to 60% (22/30); (2) good, for those who scored from 60% to 70% (23/30); (3) excellent, for those who scored higher than 85% (27/30). The time taken by each student to answer the worksheet was monitored and recorded by the researcher.

#### 3.4.2. Tool II: “Learning Abilities Feedback Questionnaire”

The questionnaire was developed to investigate the students’ subjective viewpoints about the computer-based scenarios and their influence on their general learning abilities and nursing-specific abilities. All statements were rated using the 4-point Likert scale. Responses to each item ranged from 1, strongly disagree, to 4, strongly agree [31,32,33,34].

The theoretical background used for developing this tool was the constructivist and technology acceptance approaches, which aimed to investigate nursing students’ perceptions of the use of virtual reality in managing life-support situations. It included reflective statements regarding enjoyment: an increasing motivation for learning; system quality: not yielding to the feeling of being lost, stressed, or nervous; applicability and ease of use: the authentication of learning and the application of classroom learning; usefulness: enhancing fast decision-making, demonstrating clinical reasoning skills, empowering personal control over learning, and increasing self-confidence in problem-solving and decision-making abilities [18,34]. The reflective statements listed in the nursing-specific abilities section of the questionnaire reflect all steps and phases of the nursing process using the assessment, diagnosis, planning, implementation, and evaluation (ADPIE) model [35,36,37]. It also included guiding statements from Bandura’s self-efficacy theory, such as increasing self-confidence in one’s own problem-solving and decision-making abilities [38,39,40].

### 3.5. Procedure

#### 3.5.1. Preliminary Phase

This phase started one week before the implementation of the computer-based scenarios. The aim and objective of computer-based learning were explained to the students. All students were advised to set up the application and practice on their computers at home. They received the theoretical part about the different types and management of dysrhythmias in the classroom. At the beginning of the clinical rotation, students were assigned to either the study group or the control group. Both groups were informed that they would be exposed to two case studies about dysrhythmias: one in week 3 and one in week 5. The students were also informed that they would be evaluated against the three criteria mentioned in tool one. 

#### 3.5.2. Implementation Phase

In this phase, the two groups were compared regarding decision-making skills. The decision-making skills worksheet was developed using questions at different levels of difficulty about dysrhythmias. In addition, student feedback on computer-based scenarios was investigated. 

Case study scenarios: These case scenarios were selected based on the learning objectives of the critical care nursing course from the Critical Thinking Cases in Nursing textbook, which focuses on case studies in different clinical practice areas [41]. After this, the case studies were transformed into computer-based scenarios for the study group and paper-based scenarios for the control group.

Study group: Students in the study group were prepared through orientation sessions regarding the objective of computer-based learning. Within the clinical rotation (seven weeks), each clinical instructor was responsible for one group of six students. The computer software was installed on all computers in the lab. Both case studies included changes in the hemodynamic parameters: heart rate, respiratory rate, blood pressure, oxygen saturation (SpO_2_), and end-tidal carbon dioxide (EtCO_2_). Each student was instructed to recognize the type of dysrhythmia, the hemodynamic changes in five different time intervals, and the priority of management for each type of dysrhythmia using the worksheet. Each student’s finishing time for the whole case study was recorded using a stopwatch. For the two case studies, the average mean scores were calculated. During the study, two researchers were available to be facilitators and to solve any technical issues.

Control group: Students in the control group were subjected to paper-based scenarios, which was the simulation instruction used in all previous semesters. The written case study presented the students with the patient’s medical history, medications taken, and reason for hospitalization. As the case study unfolded, a series of five hemodynamic changes were provided. All the information about the scenarios was provided, and the scores were recorded using the worksheet. 

In both groups, questions were guided by requiring students to use the decision-making model in each phase of the nursing process phases, like decision-making in relation to the urgent and more fatal complaints given in the data, decision-making in relation to the diagnosis of the case, decision-making in relation to the type of management by weighing the fatality of the symptom, putting the priority of management for the most life-threatening symptom, and also deciding the priority of the type of management to treat each symptom. Finally, the student also had to decide upon the most valued medical ways of monitoring the deterioration or progression of the case. 

#### 3.5.3. Final Phase

In the closing phase, both groups received debriefing for 15 min after each case study. The time selected for each student to complete the worksheet was about 30 min. At the end of the simulation session, the worksheets were collected. The mean submission time scores were calculated. The students’ feedback was then analyzed to investigate their viewpoints about the case scenario strategy used. 

Ethical consideration: An ethical clearance was obtained from the College of Applied Medical Sciences postgraduate and scientific research committee. Additionally, written informed consent was obtained from students after explaining the purpose of the study and prior to participation. They were informed that their participation is voluntary and they can withdraw at any time. The confidentiality, privacy, and anonymity of the students and their responses were assured through the phases of the study. Participants received no financial compensation for their participation.

Pilot Study: The tools and computer-based scenarios were piloted on ten students to assess whether they were applicable and clear; modifications were made accordingly. Piloted students were excluded from the main sample. The tools were developed and reviewed by an expert critical care nursing and nursing education panel to assess content validity. Test-retest reliability was 0.88 for Tool 1, and internal consistency was 0.79 for Tool 2.

Data Analysis: Data were examined for consistency and coherency, fitting with conflicts. Descriptive statistics were calculated to summarize the quantitative data using IBM SPSS Statistics for Windows, version 22.0 (IBM, Armonk, NY, USA). An independent t-test was used to compare the mean scores of the study group and the control group’s decision-making skills and to compare the mean scores of general learning abilities and nursing-specific abilities.

## 4. Results

**Figure 3:**
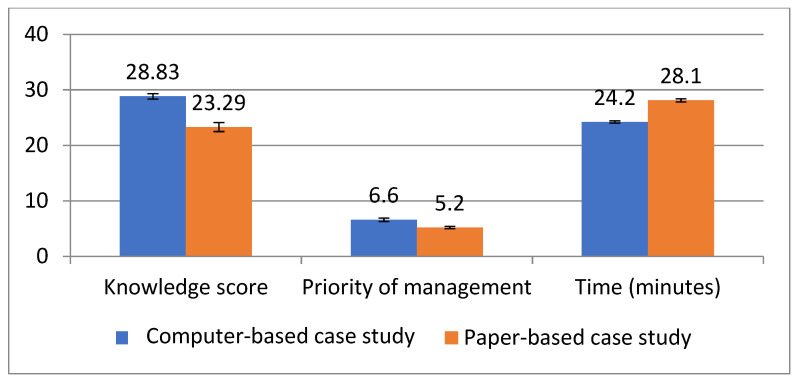
Comparison between study and control groups regarding decision-making worksheet.

Figure 3 displays the results of the decision-making skills worksheet and a comparison between the study groups (computer-based case study) and the control groups (paper-based case study). In terms of the knowledge score, the mean score in the study group was (28.83 ± 0.5), which was significantly higher than the control group (23.29 ± 0.8) (*p* = 0.05). Additionally, there was a significant difference between both groups regarding the time spent in submitting the case studies, as it was completed faster in the computer-based case study group compared to the paper-based group (*p* = 0). On the other hand, there was no statistically significant difference between the two study groups in answering the management priority of dysrhythmias.

Table 1 displays nursing students’ feedback about computer-based case study regarding their general learning and nursing-specific abilities. When answering questions about the general learning abilities, most of the students (98.7%) who were exposed to a computer-based case study had positive feedback about enhancing fast decision-making and empowering control over learning, respectively. Additionally, these students did not yield the feeling of stress, nervousness, and the feeling of being lost.

Concerning nursing students’ abilities, 98.7% of students had positive feedback about increasing critical analysis ability to reach a nursing diagnosis, fostering patient response evaluation, and understanding the individual’s entire complex situation, respectively.

Table 2 shows the total mean scores of students’ feedback from the computer-based simulation group. It was found that the total mean score of general learning abilities was 3.16 ± 0.26, whereas the total mean score of nursing-specific abilities was 3.71 ± 0.37, which was found to be statistically significant (*p* = 0).

## 5. Discussion

Computer-based learning is a term used for any kind of learning using computers to deliver instructional strategies to meet the students’ needs. The educational strategy is significant, as it provides training that is similar to practicing with real systems but at a low cost and greater safety for patients and students. Incorporating computer-based simulation in nursing education courses is not a matter of luxury, especially in life support and end-of-life care. Computer-based training allows students to learn assessment techniques and clinical skills in an authentic environment, so they can begin to develop clinical decision-making and prioritization skills. Students also start to become familiar with the complex healthcare environment with the knowledge required to provide safe patient care [42,43].

The decision-making performance of the students is reflected by the knowledge score, the time it took to complete the case study, and the mean score of prioritizing patient management with each type of dysrhythmias. The findings are in favor of the computer-based simulation group, meaning that the students had higher knowledge scores, spent less time completing the case study, and had better scores in prioritizing care management. A study conducted by Padilha et al. (2019) proved that students’ clinical reasoning and knowledge improved using virtual clinical simulations [3]. As the computer software was readily available in the students’ computers at home, they practiced more frequently and received more benefits in the current study. Foronda et al. 2020 confirmed in their systematic review of 69 studies that the amount the time spent in virtual simulation correlated with greater learning benefits [44]. In line with that were the results of seven studies that were reported by Chen et al., who reported that virtual reality education could improve the knowledge of participants more effectively than the traditional teaching methods. The current study also resembled this study as there was a significant improvement in knowledge, satisfaction, and confidence of nursing students in using computer technology in teaching [45].

On the other hand, in the randomized controlled trial of Cobbett and Snelgrove-Clarke, there were no statistically significant differences found in students’ knowledge when comparing face-to-face learning and virtual simulations [46]. Moreover, the current study showed high, significant differences between both groups regarding the time spent decision-making. This contradicts the William et al. study [47].

The nursing profession, generally, is centered around ADPIE. Clinical reasoning and decision-making are considered crucial for the nurse who guides each phase [37]. The present research showed high level mean scores regarding such a strategy, which is parallel to the results of Sattar M., 2019, who found an overwhelming preference for such a strategy over traditional learning as it can motivate and improve the competency of students [48].

In comparing their viewpoints regarding developing general skills that any student must possess and nursing-specific skills, the current research showed that it is most suitable for teaching nursing skills and nursing courses. Indeed, most of the students’ feedback (above 94%) regarding the general abilities acquired through computer-based simulation was positive.

Most of the students participating in the current study supported that the computer software enhanced their understandings of the future professional role of a nurse. The software improved the ability to link concepts and principles from nursing and other sciences when making decision-making. This included ‘critical life’ scenarios and conflict management. All of these nursing-related activities demonstrated positive outcomes, such as the development of competencies related to leadership and management skills. In this regard, Kiernan L., 2018 emphasized the importance of preparing students for a complex healthcare environment and providing safe patient care [43].

The current research revealed that students ensured that they developed an understanding of the complexity of care and tried to be aware of the individual’s entire situation since it increased their critical analysis ability. Understandings of the situation allowed students to extract information through assessment data that helped them to reach suitable nursing diagnoses. Huang and Liaw’s (2018) qualitative study supported the notion that virtual reality could help nursing students to create a highly intuitive and interactive user experience [18]. In 2019, Hannans and Nevins added that computer-based scenarios give a more in-depth understanding of disease processes [49]. Similarly, Nibbelink et al. (2018) concluded that computer software provides an unlimited opportunity for nursing education, creating a realistic, non-threatening environment to practice clinical decision-making and practical skills at no risk to patients [6].

Moreover, the majority of students confirmed that computer-based case studies foster a patient response evaluation for earlier recognition of any deterioration in the entire condition. According to this instance and as expressed by participant nursing students on verbatim, scenario-based simulation not only improved assessment skills for the acute patients but also improved their abilities to recognize deteriorating patients [50].

The study also revealed that, as reported by more than 94% of students who engaged in computer-based simulation learning strategy and as reported in the literature and previous studies, this strategy succeeded in enhancing fast decision-making, applying class-gained learning, fostering feelings of student empowerment and control over learning, and increasing self-confidence as well as motivation and overall positive learning experience. At the same time, students did not report negative impressions from the new strategy, such as the feelings of being lost or stressed and nervous [51,52,53,54].

Conversely, the study of Saab et al. (2021) indicated that if students report a lack of interest in virtual reality, it may result in some students becoming distracted and disengaged [55]. This incongruence with our results in relation to satisfaction might be related to the immaturity of the technology design used in other studies or even the high expectations of students in Western countries rather than in the Middle East.

## 6. Limitations of the Study

The current study encountered few limitations. To begin, the sample was limited to only one course (Critical Care Nursing). This study needs to be replicated on future students enrolled in the same course and applied to other courses that require decision-making skills in the nursing or medical fields to give greater validity to the findings. The control group size was relatively small in comparison to the study group. Decision-making skills were only measured after the implementation of the simulation strategies, and it would perhaps be better to measure both before and after implementation.

## 7. Conclusions and Recommendations

The findings of this study indicate that the students who used computer-based simulations of life-threatening events, such as patients with different diagnoses of dysrhythmias, reached higher achievement scores in their case scenarios in a shorter time compared to the traditional method (paper-based case scenario). The decision-making skills can be found in their precise answers, the time it took to finish the case study, and the priority of management of the case. In addition, the students exposed to computer-based scenarios reported positive feedback regarding their general and nursing-specific abilities, which will enhance the tendency to use such technology in clinical teaching in the future.

## Figures and Tables

**Table 1:** Frequency distribution of a computer-based case study group regarding their feedback on learning abilities (*n* = 78).

Feedback of Learning Abilities	Computer-Based Case Study	
	No. of Students with Positive Feedback (SA and A)	No. of Students with Negative Feedback (SD and D)
	No. (%)	No. (%)
General Learning Abilities		
Increasing motivation for learning	74 (94.4%)	4 (5.1%)
Demonstrating clinical reasoning skills	73 (94.6%)	5 (5.4%)
Authentication of learning	72 (92.3%)	6 (7.7%)
Enhancing fast decision-making	77 (98.7%)	1 (1.3%)
Application of class-gained learning	75 (96.1%)	3 (3.9%)
Not yielding feeling of stress and nervousness	77 (98.7%)	1 (1.3%)
Empowering control over learning	77 (98.7%)	1 (1.3%)
Increasing self-confidence in problem-solving and decision-making abilities	75 (96.1%)	3 (3.9%)
Not yielding the feeling of being lost	77 (98.7%)	1 (1.3%)
Nursing-Specific Abilities		
Deepening of knowledge about medical diagnosis and nursing case management	76 (97.4%)	2 (2.6%)
Improving understanding of the complexity of nursing care	75 (96.1%)	3 (3.9%)
Gaining understanding of future professional role	75 (96.1%)	3 (3.9%)
Increasing critical analysis and ability to reach nursing diagnosis	77 (98.7%)	1 (1.3%)
Prioritizing nursing diagnosis scientifically	76 (97.4%)	2 (2.6%)
Individualizing each case nursing interventions	76 (97.4%)	2 (2.6%)
Fostering patient response evaluation	77 (98.7%)	1 (1.3%)
Linkage of concepts and principles from nursing and other sciences in clinical decision-making	76 (97.4%)	2 (2.6%)
Recognizing patient deterioration early	72 (92.3%)	6 (7.7%)
Understanding the individual’s entire complex situation	77 (98.7%)	1 (1.3%)
Recognizing holistic perspective of the patient’s life world	75 (96.1%)	3 (3.9%)

SA: Strongly Agree, A: Agree, SD: Strongly Disagree, D: Disagree.

**Table 2:** Total mean scores of students’ feedback regarding computer-based simulation group. (*n* = 78).

Total Feedback Score	Mean	SD	*t*-Test (*p*)
General learning abilities	3.16	0.26	88 (0 *)
Nursing-specific abilities	3.71	0.37	

* *p*: significant if <0.05.

## Data Availability

The data presented in this study are available within the article.
